# Canopy structure of tropical and sub-tropical rain forests in relation to conifer dominance analysed with a portable LIDAR system

**DOI:** 10.1093/aob/mct242

**Published:** 2013-11-05

**Authors:** Shin-ichiro Aiba, Kosuke Akutsu, Yusuke Onoda

**Affiliations:** 1 Graduate School of Science and Engineering, Kagoshima University, Kagoshima 890-0065, Japan; 2 Graduate School of Environmental Science, Hokkaido University, Sapporo 060-0810, Japan; 3 Graduate School of Agriculture, Kyoto University, Kyoto 606-8502, Japan

**Keywords:** Additive basal area, altitudinal gradient, canopy structure, conifer–angiosperm coexistence, forest stratification, portable LIDAR system, Mount Kinabalu, Yakushima Island

## Abstract

**Background and Aims:**

Globally, conifer dominance is restricted to nutient-poor habitats in colder, drier or waterlogged environments, probably due to competition with angiosperms. Analysis of canopy structure is important for understanding the mechanism of plant coexistence in relation to competition for light. Most conifers are shade intolerant, and often have narrow, deep, conical crowns. In this study it is predicted that conifer-admixed forests have less distinct upper canopies and more undulating canopy surfaces than angiosperm-dominated forests.

**Methods:**

By using a ground-based, portable light detection and ranging (LIDAR) system, canopy structure was quantified for old-growth evergreen rainforests with varying dominance of conifers along altitudinal gradients (200–3100 m a.s.l.) on tropical and sub-tropical mountains (Mount Kinabalu, Malaysian Borneo and Yakushima Island, Japan) that have different conifer floras.

**Key Results:**

Conifers dominated at higher elevations on both mountains (Podocarpaceae and Araucariaceae on Kinabalu and Cupressaceae and Pinaceae on Yakushima), but conifer dominance also varied with soil/substrate conditions on Kinabalu. Conifer dominance was associated with the existence of large-diameter conifers. Forests with higher conifer dominance showed a canopy height profile (CHP) more skewed towards the understorey on both Kinabalu and Yakushima. In contrast, angiosperm-dominated forests had a CHP skewed towards upper canopy, except for lowland dipterocarp forests and a sub-alpine scrub dominated by small-leaved *Leptospermum recurvum* (Myrtaceae) on Kinabalu. Forests with a less dense upper canopy had more undulating outer canopy surfaces. Mixed conifer–angiosperm forests on Yakushima and dipterocarp forests on Kinabalu showed similar canopy structures.

**Conclusions:**

The results generally supported the prediction, suggesting that lower growth of angiosperm trees (except *L. recurvum* on Kinabalu) in cold and nutrient-poor environments results in a sparser upper canopy, which allows shade-intolerant conifers to co-occur with angiosperm trees either as emergents or as codominants in the open canopy.

## INTRODUCTION

Globally, conifer dominance is restricted to marginal habitats at high altitudes and latitudes, on nutrient-poor or inundated soils, and in dry lands (Bond, 1989; Becker, 2000; Farjon, 2008). In East Asia, temperate coniferous forests consisting of the families Pinaceae and Cupressaceae occur on mountains at the transitional zone between sub-tropical and cool-temperate zones, while sub-alpine and boreal coniferous forests dominated by Pinaceae occur at higher altitudes and latitudes (Ohsawa, 1993). In tropical South-east Asia and New Guinea, conifers of Podocarpaceae and Araucariaceae often become dominant at high altitudes and on infertile or waterlogged soils in lowland (Enright and Jaffré, 2011). From this global pattern, we can generalize that conifers are associated with infertile soils in colder, drier or waterlogged environments where the decomposition rate is slower (Grubb, 1977; Swift *et al.*, 1979).

Conifer dominance may be determined by competition with angiosperms rather than by environmental filtering because conifers can grow well on fertile soils beyond their natural range when planted (Bond, 1989). Bond's (1989) slow seedling hypothesis postulates that conifers inherently grow more slowly on fertile soils than do angiosperm trees because of their less efficient transport system (non-reticulated leaf veins and tracheids), and consequently they can outcompete angiosperms only where growth of angiosperms is limited. Above-ground competition for light in the regeneration niche plays the central role in this hypothesis because conifer seedlings die (or may not even germinate) under the deep shade of an angiosperm canopy on fertile soils (Becker, 2000; Coomes and Bellingham, 2011). Generally, conifers in temperate East Asia and tropical South-east Asia are shade intolerant (Suzuki and Tsukahara, 1987; Tang and Ohsawa, 2002; Enright and Jaffré, 2011; Kitayama *et al.*, 2011) as are several species in the western Pacific including New Zealand, New Guinea and New Caledonia (Enright *et al.*, 1999; Coomes *et al.*, 2005; Carswell *et al.*, 2012).

Analysis of canopy structure is important for understanding the mechanism of plant coexistence in relation to competition for light. Poor growth of angiosperm trees on poor soils or in cold environments will result in a sparse upper canopy composed of widely spaced crowns of angiosperm trees. A sparse upper canopy will facilitate shade-intolerant conifers to regenerate successfully in spaces between angiosperm crowns or in canopy gaps formed by small-scale disturbance (e.g. tree fall). Enright *et al.* (1999) concluded that this mechanism maintained low density stands of shade-intolerant conifer under open canopies in the absense of large-scale distrubance (e.g. fires). In such open-canopied forest, conifers may codominate with angiosperm trees in the upper canopy, with little differentiation in canopy heights. Many conifers inherently have narrow, deep, conical (or cylindrical) crowns (Walcroft *et al.*, 2005; Farjon, 2008; Lusk *et al.*, 2012), which contributes to the ‘erect’ canopy structure of mixed conifer–angiosperm forests where foliage is less concentrated in the upper canopies (Koike *et al.*, 1990). Mixed forest will also show an undulating outer canopy surface because of widely spaced crowns including the conical crowns of conifer. In contrast, angiosperm trees usually have broad, shallow, spherical crowns, which results in a distinct upper canopy layer with a smooth outer surface in angiosperm forests (Koike and Hotta, 1996).

However, in closed-canopy stands where above-ground competition for light is intense, shade-intolerant conifers may become emergent above the general forest canopy comprising more shade-tolerant angiosperm trees. The ‘additive basal area’ phenomenon, where stand basal area (a surrogate of above-ground biomass) of mixed conifer–angiosperm forest becomes greater than that of angiosperm-dominated forest, has been reported for temperate and tropical evergreen forests especially in the Southern Hemisphere (Enright and Ogden, 1995). Differential utilization of light by taller conifers and shorter angiosperm trees that have different above-ground architectures has been suggested as the cause of additive basal area, although below-ground resource partitioning and the lack of disturbance may also be involved (Enright, 1982; Ogden, 1985; Enright and Ogden, 1995; Lusk, 2002; Midgley *et al.*, 2002; Lawes *et al.*, 2006; Aiba *et al.*, 2007). If this suggestion holds, mixed forest should show multiple peaks in vertical distribution of foliage and highly undulating canopy surface corresponding to vertical niche partitioning between conifers and angiosperms.

Thus, it can be predicted that conifer-admixed forest, generally occurring at higher altitudes or latitudes and/or on poorer soils, should have less distinct upper canopies and more undulating canopy surfaces than angiosperm-dominated forests (or broadleaf forests). To test this prediction, we quantify canopy structure of evergreen rainforests with varying dominance of conifers along altitudinal gradients and on different soil/substrates. Our study sites encompass a broad range of mean annual temperature equivalent to a latitudinal gradient from equatorial to cool-temperate forests. To explore general trends in coexistence between conifers and angiosperm trees, we compare two mountains that have different conifer floras. One is a tropical mountain (Kinabalu, Borneo) dominated by southern conifers (Podocarpaceae and Araucariaceae), and the other is a sub-tropical mountain (Yakushima, Japan) dominated by predominantly northern conifers (Pinaceae and Cupressaceae).

Due to technical difficulties, canopy structure has usually been studied in a single forest (e.g. Koike and Syahbuddin, 1993; Fukushima *et al.*, 1998). Few studies have compared canopy structure of many forests across climatic gradients (e.g. Koike *et al.*, 1990; Koike and Hotta, 1996), and none addressed the issue of conifer–angiosperm coexistence. We used a ground-based, portable light detection and ranging (LIDAR) system developed by Parker *et al.* (2004) to quantify above-ground forest structure along altitudinal gradients. This system enables us to determine both vertical distribution of canopy area and canopy surface topography with less labour than other ground-based, camera methods (e.g. Koike and Syahbuddin, 1993; Fukushima *et al.*, 1998; Birnbaum, 2001). All our study sites are in very wet rain forests where rainfall and fog are frequent and unpredictable, and this system has advantages in collecting many data efficiently in difficult field conditions. Also our system costs much less than an airborne LIDAR system (e.g. Clark *et al.*, 2004; Asner *et al.*, 2009) although it is not suited to survey extensive areas. Our study sites show the variation of forest structure reflecting local soil/substrate conditions at similar altitudes, and a smaller-scale, ground-based method would be more appropriate.

## MATERIALS AND METHODS

### Study sites

Mount Kinabalu (6 °N, 116 °E), Malaysian Borneo, is the highest mountain (summit elevation 4095 m a.s.l.) in South-east Asia between New Guinea and the Himalayas (Supplementary Data Fig. S1). This mountain shows a typical tropical vegetation zonation exclusively composed of evergreen trees (Kitayama, 1992; Ohsawa, 1993). The geological substrates are dominated by Tertiary sedimentary rock below approx. 3000 m and by granite above that. Ultrabasic rock and unconsolidated Quaternary sediments are distributed as patches at some elevations. Notably, soil fertility is lower on ultrabasic rock than on the other types of substrate at the same elevation (Kitayama and Aiba, 2002). Diverse forests reflecting varied altitude and geology are protected within a 754 km^2^ area of Kinabalu Park. Mean annual rainfall is about 2500 mm throughout the slope. Mean annual temperature *T* (°C) decreases linearly with increasing altitude *A* (m): *T* = 27·5 – 0·0055*A* (Kitayama, 1992).

Yakushima (30 °N, 130 °E), southern Japan, with an area of 504 km^2^, is an island that has the highest mountain (Mount Miyanouradake, summit elevation 1936 m a.s.l.) between mainland Japan and Taiwan. Yakushima can be regarded as the northernmost mountain that shows a tropical type of vegetation zonation dominated by evergreen trees throughout the slope, though winter-deciduous broadleaf species also occur, and the forests there may be called sub-tropical (Ohsawa, 1993). The geological substrate of the massif is granite, and Tertiary sedimentary rocks are distributed in the lowland below approx. 300 m, except in the north-western part where granite occurs throughout the flank. Mean annual rainfall is 2400–5900 mm in lowland and reaches as high as 7400 mm in the inland mountainous area. Mean annual temperature decreases linearly upslope: *T* = 19·8 – 0·0061*A* (Aiba *et al.*, 2007).

Dominant conifers are *Dacrycarpus* spp., *Dacrydium* spp., *Falcatifolium falciforme*, *Podocarpus gibbsii*, *Phyllocladus hypophyllus* (Podocarpaceae) and *Agathis* spp. (Araucariaceae) on Kinabalu (Kitayama, 1992; Aiba and Kitayama, 1999; Aiba *et al.*, 2007; Kitayama *et al.*, 2011), and *Cryptomeria japonica* (Cupressaceae), *Abies firma* and *Tsuga sieboldii* (Pinaceae) on Yakushima (Irikura, 1984; Ohsawa, 1984; Suzuki and Tsukahara, 1987; Aiba *et al.*, 2007; Ishii *et al.*, 2010). Dominant angiosperm trees belong to Dipterocarpaceae and Myrtaceae in lowland and montane (≥1200 m) forests, respectively, on Kinabalu, and to Hamamelidaceae (*Distylium racemosum*), Fagaceae and Lauraceae on Yakushima, the latter two families also being well represented in Kinabalu montane forests.

### Field methods, data and analysis

The LIDAR system used in this study (LD90-3100HS, Riegl Laser Measuement Systems, Horn, Austria) measures the vertical distance to plant surfaces (leaves, branches, trunks, etc.) at many points rapidly by emitting laser pulses about 200 times per second (Parker *et al.*, 2004). By applying the method of MacArthur and Horn (1969), the vertical profile of the plant surface, which is termed the canopy height profile (CHP) by Parker *et al.* (2004), was calculated. The MacArthur and Horn method does not yield the absolute leaf area index (LAI) but provides relative LAI at a given height interval (Aber, 1979), which we call the relative CHP. Relative CHP determination by the MacArthur and Horn method has not been rigorously validated for conifer canopy, but it has yielded reasonable results for mixed conifer forest in western North America (Lefsky *et al.*, 1999).

We selected ten plots on Kinabalu and nine plots on Yakushima (Table 1). Mean annual temperature varies from 10 to 24 °C among our study plots, equivalent to a latitudinal range from equatorial to cool-temperate (approx. 40–50 °N) lowland forests. All the forests are old growth without human disturbance for >100 years. Plot size varied, but all plots were rectangular in plan view and the length of the shorter sides was greater than the maximum canopy height. All trees ≥5 cm diameter at breast height or 1·3 m above-ground (DBH) were measured for DBH and identified to species. Tree height, i.e. the vertical distance from the trunk base to the crown top, was measured for >50 trees per plot, including seemingly the tallest ones by using a clinometer. For the LIDAR measurements, we established three 100 m or five to six 50 m lines separated by 10 m in each plot, yielding total line lengths of 250–300 m for each plot. In the K31U plot (30 m × 20 m) on Kinabalu, five 50 m lines parallel to the longer sides of the plot and separated by 5 m were expanded outside the plot. We avoided foggy weather for measurement as we found that this system responded to fog.

**Table 1.** Elevation, vegetation zone, geological substrate, plot area, maximum tree height, mean annual temperature (MAT) and abbreviation of the study plots

Site and elevation (m)	Vegetation zone	Geological substrate	Plot area (ha)	Max. tree height (m)	MAT (°C)	Abbreviation
Kinabalu						
650	TLF	Sedimentary rock	1·00	46·8	23·9	K07S
700	TLF	Ultrabasic rock	1·00	65·4	23·7	K07U
1560	TLMF	Sedimentary rock	0·50	30·0	18·9	K17S
1860	TLMF	Ultrabasic rock	0·20	22·6	17·3	K17U*
1860	TLMF	Quaternary	1·00	32·1	17·3	K17Q
1950	TLMF	Ultrabasic/Quaternary	0·50	35·7	16·8	K17UQ*
2590	TUMF	Sedimentary rock	0·25	20·6	13·3	K27S
2700	TUMF	Ultrabasic rock	0·20	14·2	12·7	K27U*
3050	TSF	Ultrabasic rock	0·06	15·0	10·7	K31U
3080	TSF	Granite	0·20	6·1	10·6	K31G*
Yakushima						
170	WTBF	Sedimentary rock	0·50	23·3	18·8	Y02Sa
200	WTBF	Sedimentary rock	0·25	19·3	18·6	Y02Sb
230	WTBF	Sedimentary rock	0·20	NA†	18·4	Y02Sc
280	WTBF	Granite	0·50	19·8	18·1	Y02G
570	WTBF	Granite	0·50	22·3	16·3	Y06Ga
600	WTBF	Granite	0·25	NA†	16·1	Y06Gb
1050	CTMF	Granite	0·25	33·8	13·4	Y12Ga*
1200	CTMF	Granite	0·50	33·1	12·5	Y12Gb*
1550	CTMF	Granite	0·25	22·3	10·3	Y16G*

Vegetation zone is based on Kitayama (1992) for Kinabalu, and Ohsawa (1984) for Yakushima.

TLF, tropical lowland forest; TLMF, tropical lower-montane forest; TUMF, tropical upper-montane forest; TSF, tropical sub-alpine forest/scrub; WTBF, warm-temperate broadleaf forest; CTMF, cool-temperate mixed conifer–broadleaf forest).

For geological substrate, Quaternary indicates fine-grained Quaternary sediments and Ultabasic/Quaternary indicates ultrabasic rock overlain by rocky Quaternary sediments.

MAT was esitimated by the equations of Kitayama (1992) and Aiba et al. (2007).

*Mixed forests with conifer relative basal area >25 %.

†Data not available.

Parker *et al.* (2004) walked straight lines at a constant speed for measurement, but this was often impractical in the difficult terrain on the mountain slopes. We thus took measurements at 2 m intervals along lines, by swinging the system half-way around the body at each point (Akutsu *et al.*, 2007). The distance was converted to height above ground by adding 1 m (the approximate height of the system at the measurement). We then grouped the data in horizontal bins for every 50 m line. Shorter bins were insufficient in many of our study forests to acquire some sky observations (resulting from laser passing through the canopy without hitting the plant surface) that are necessary for the procedure of MacArthur and Horn (1969). Even the 50 m bins sometimes failed to sample sky observations, and such bins were excluded from analysis, leaving five or six bins for each plot (except Y02Sa and Y02Sc plots where only four and three bins were left, respectively). As an alternative approach, we added one sky observation to bins in cases without sky observation to calculate relative CHP for bins of 2 and 10 m length and confirmed that similar results were obtained (Supplementary Data Fig. S2). Relative CHP at 1 m height intervals of each bin was averaged for each plot to obtain plot-level relative CHP. The shape of plot-level relative CHP was characterized by the coefficient of skewness (CS; a measure of asymmetry) by using midpoint height (1·5m, 2·5 m, and so on) to represent each height class.

**Fig. 1.**
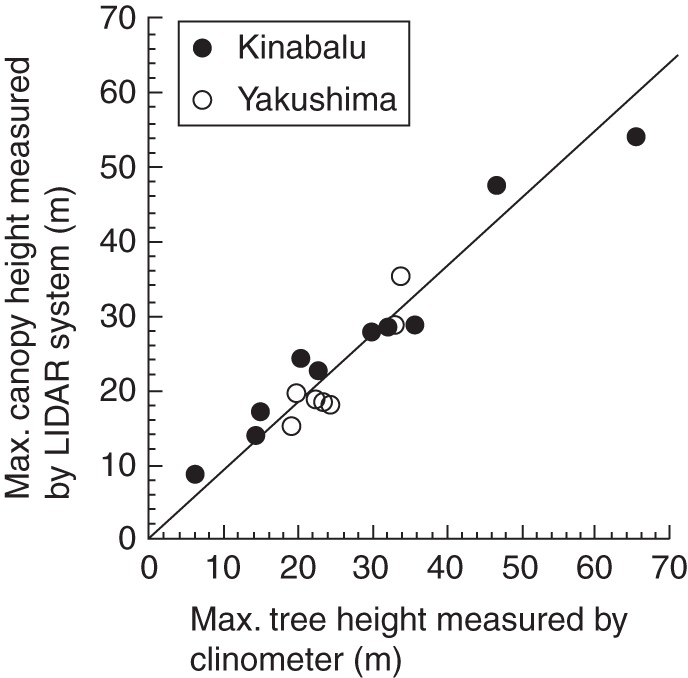
The relationship between maximum tree height measured by a clinometer and maximum canopy height measured by the LIDAR system among the study plots. The line represents standardized major axis regression forced through the origin (the intercept was not significant at *P* < 0·05): *Y* = 0·90*X* (*R*^2^ = 0·93, *P* < 0·001, 95 % confidence intervals of slope: 0·84–0·96).

We also examined the maximum canopy height recorded at each point, or local outer canopy height (LOCH), assuming that the LIDAR system could sample most heights overhead (Parker *et al.*, 2004). This assumption was satisfied because maximum canopy heights measured by LIDAR for each plot were similar to maximum tree heights measured by a clinometer, with slightly lower estimations in LIDAR measurements (Fig. 1). Thus, we can conclude that LIDAR measurements reproduced altitudinal patterns in maximum tree height on both mountains reasonably well. We characterized the horizontal heterogeneity of the canopy surface by calculating the coefficient of variation (CV; standard deviation divided by the mean) of LOCH for each plot.

## RESULTS

### Conifer dominance

**Fig. 2.**
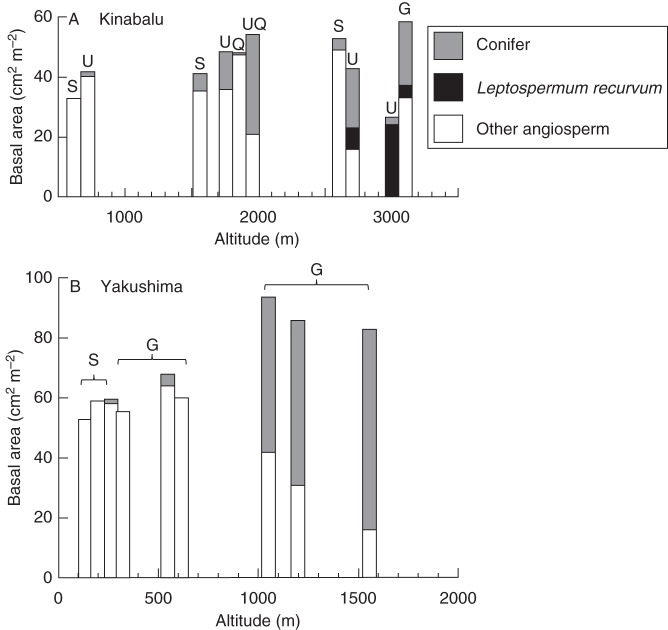
Altitudinal change of basal area (≥5 cm DBH) of conifer, *Leptospermum recurvum* (Myrtaceae) and other angiosperm trees in the study plots on: (A) Mount Kinabalu, Borneo and (B) Yakushima Island, Japan. Note that the scale of the horizontal axis differs between the two panels. Geological substrates are shown for each plot: G, granite; S, sedimentary rock; Q, fine-grained Quaternary sediments; U, ultrabasic rock; UQ, ultrabasic rock overlain by rocky Quaternary sediments.

Dominance of conifer in terms of basal area generally increased with increasing altitude on Kinabalu, but also depended on local soil/substrate conditions (Fig. 2A). For example, in the lower montane zone at 1560–1950 m a.s.l., conifer dominance increased in the order of fine-grained Quaternary sediments (K17Q) < sedimentary rock (K17S) < ultabasic rock (K17U) < ultrabasic rock overlain by rocky Quaternary sediments (K17UQ). Similarly, forests on ultrabasic rock showed higher dominance of conifer than those on sedimentary rocks in the lowland zone at 650–700 m and in the upper montane zone at 2590–2700 m. Sub-alpine forests at 3050–3080 m are exceptions; forest on ultrabasic rock (K31U) showed the monodominance of a small-leaved broadleaf tree *Leptospermum recurvum* (Myrtaceae), almost lacking conifers. Elsewhere on Kinabalu, *L. recurvum* dominates on shallow soils in the summit region at >3300 m on granite (Smith, 1980). Therefore, the dominance of trees shifted from *L. recurvum* to conifers and then to other broadleaf trees from harsher to milder environments across a combined gradient of altitude and soil/substrate conditions on Kinabalu.

The basal area of conifers sharply increased from 600 to 1050 m with increasing altitude on Yakushima (Fig. 2B). The increase of conifers more than offset the decrease of broadleaf trees so that the basal area of conifers appeared to be additive at high elevations at ≥1050 m according to the model of Enright and Ogden (1995). The difference in geological substrates (sedimentary rock vs. granite) did not affect the dominance of conifer in the lowland ≤600 m on Yakushima.

On both mountains (except the K31U plot), conifer basal area was mostly accounted for by large-diameter stems, indicating vertical niche segregation between overstorey conifers and understorey angiosperms (Supplementary Data Fig. S3). This was especially manifest in the mixed forests where the relative basal area of conifers was >25 % (Table 1).

### Canopy height profile

**Fig. 3.**
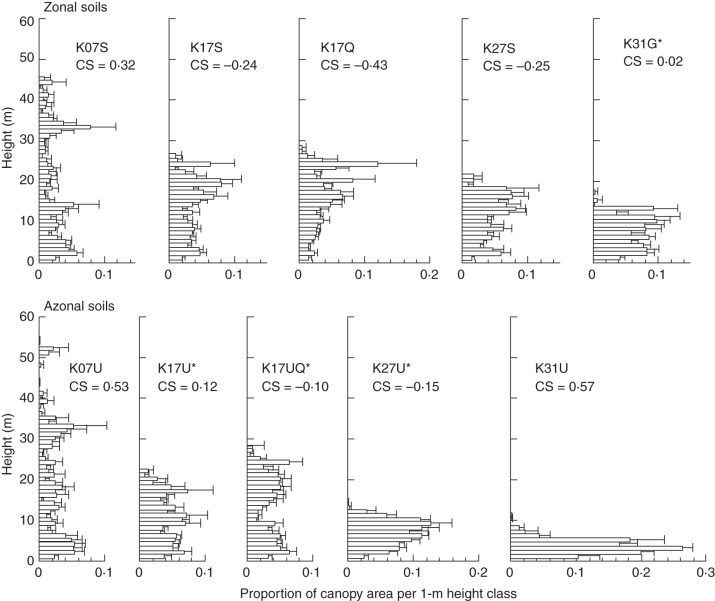
Relative canopy height profiles for 50 m bins across altitudinal gradients on zonal soils on sedimentary rock, Quaternary sediments and granite (upper panels) vs. azonal soils underlain by ultrabasic rocks (lower panels) on Mount Kinabalu. Horizontal bars and lines represent the mean and standard errors, respectively. Plots with asterisks are mixed forests with conifer relative basal area >25 %. The coefficient of skewness (CS) is shown for each plot.

Relative CHPs showed different altitudinal patterns between the two mountains. On Kinabalu, maximum canopy height decreased with increasing elevation, especially on azonal soils (Fig. 3). The two lowland forests dominated by dipterocarps (K07S and K07U) showed multiple peaks across the vertical profile and the overall distributions were skewed towards the understorey (i.e. CS values were distinctly positive). The montane forests ≥1560 m tended to show fewer peaks, and mixed conifer–broadleaf forests showed sparser upper canopy and denser understorey (more positively skewed distributions) than broadleaf forests (except the K31U plot). Among the montane mixed forests, the CHP of the K17UQ plot was distinctively bimodal.

**Fig. 4.**
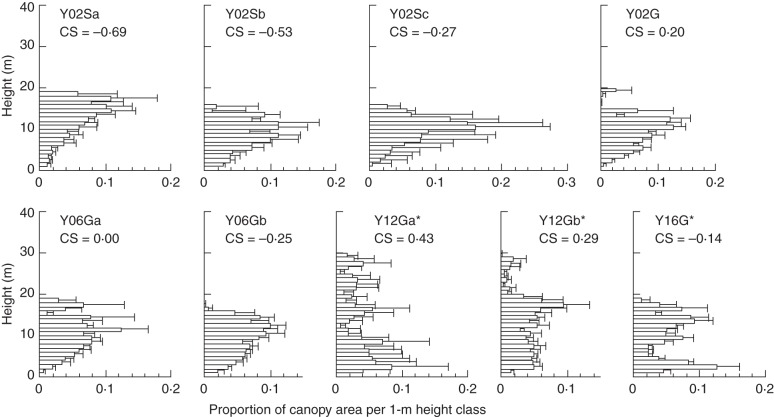
Relative canopy height profiles for 50 m bins across altitudinal gradient on Yakushima Island. See Fig. 3 for the explanation of the figure.

On Yakushima, maximum canopy height showed a monomodal pattern across altitude, two mixed forests at 1050–1200 m a.s.l. (Y12Ga and Y12Gb) showing the greatest maximum heights (Fig. 4). The relative CHP of these two forests was similar to that of the two lowland dipterocarp forests on Kinabalu in that they showed multiple peaks and highly positive CS. The mixed forest near the forest limit (Y16G) had a bimodal CHP similar to that of the K17UQ plot. Broadleaf forests ≤600 m on Yakushima showed CHPs skewed towards the upper canopy (CS was negative to moderately positive) similar to montane broadleaf forests on Kinabalu. In particular, some lowland forests (e.g. Y02Sa and Y02Sb) had distinct upper canopies showing highly negative CS.

**Fig. 5.**
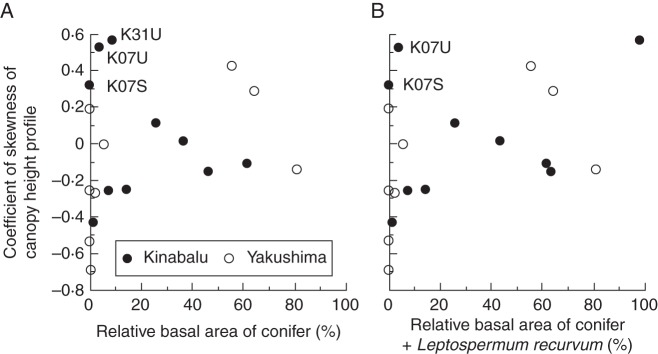
Coefficient of skewness of the canopy height profile in the study plots in relation to: (A) relative basal area of conifers and (B) combined relative basal area of conifers and *Leptospermum recurvum*. Outliers explained in the text are indicated by their plot abbreviations.

When two lowland dipterocarp forests (K07S and K07U) and a sub-alpine scrub dominated by *L. recurvum* (K31U) on Kinabalu were excluded, the CS of relative CHP became more positive with increasing dominance of conifers (Fig. 5A, *R* = 0·52, *P* = 0·04). When *L. recurvum* was treated as a conifer because of the functional similarities (i.e. small leaves and the association with the nutrient-poor habitats), the correlation became stronger even when the K31U plot was included (Fig. 5B, *R* = 0·63, *P* = 0·006).

### Canopy surface heterogeneity

**Fig. 6.**
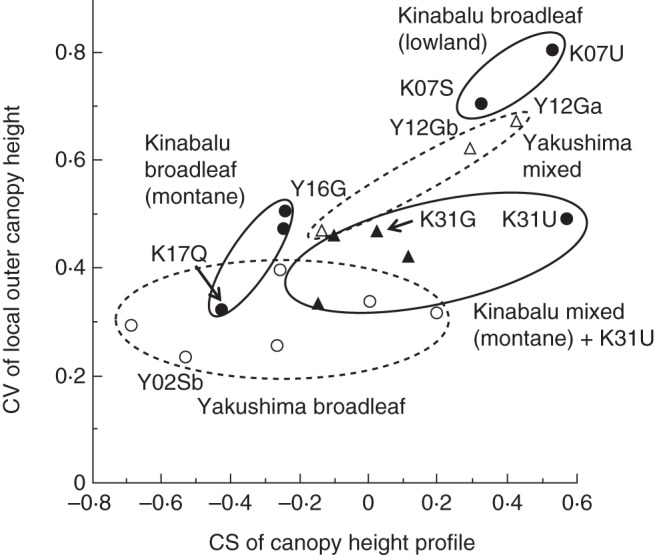
Relationship between the coefficient of skewness (CS) of the canopy height profile and the coefficient of variation (CV) of the local outer canopy height among the study plots. Filled and open symbols represent Kinabalu and Yakushima plots, respectively: circles, broadleaf forest; triangles, mixed conifer–broadleaf forest. Three groups of forests on Kinabalu are indicated by ellipses with a solid outline, and two groups on Yakushima by ellipses with a dashed outline. Plots explained in the text or shown in Fig. 7 are indicated by their abbreviations.

**Fig. 7.**
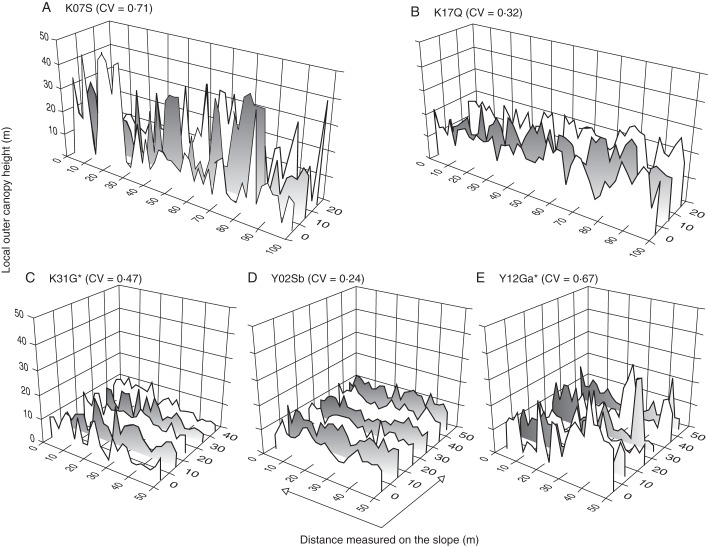
Examples of the horizontal distribution of local outer canopy height in the study plots on: (A–C) Mount Kinabalu and (D–E) Yakushima Island. The coefficient of variation (CV) is shown for each plot. Plots with asterisks are mixed forests with conifer relative basal area >25 %.

A measure of horizontal heterogeneity of the canopy surface (the CV of LOCH) was significantly positively correlated with the CS of CHP among the study plots (Fig. 6, *R* = 0·74, *P* < 0·001). This indicates that forest with a sparser upper canopy also showed a more heterogeneous distribution of outer canopy heights (examples in Fig. 7). However, there were substantial variations around the mean trend, and we can recognize three groups of forests on Kinabalu and two groups on Yakushima (Fig. 6). Two lowland diptrocarp forests on Kinabalu showed more undulating canopy surface (i.e. greater CV of LOCH) than eight montane forests. Among the eight montane forests on Kinabalu, broadleaf forests and mixed forests showed similar horizontal heterogeneity. These two forest types differed in CS of CHP, which was lower in broadleaf forests than in mixed forests (except the K31U plot). The sub-alpine scrub on ultrabasic rock dominated by *L. recurvum* (K31U) was more similar to mixed forests than to other broadleaf forests. On Yakushima, three montane mixed forests showed more heterogeneous canopy surfaces (greater CV of LOCH) than did the lowland broadleaf forests, though the mixed forest near the forest limit (Y16G) showed a relatively homogeneous canopy surface. Two lowland broadleaf forests on Kinabalu (K07S and K07U) and two montane mixed forests well below the forest limit on Yakushima (Y12Ga and Y12Gb) showed similar canopy structures with highly undulating canopy surfaces (CV of LOCH >0·6), indicating the existence of emergent layers.

## DISCUSSION

### Canopy structure and conifer dominance

Our prediction, that conifer-admixed forests at higher elevations and/or on poorer soils had less distinct upper canopies and more undulating canopy surfaces than angiosperm-dominated forests, was generally supported for both tropical and sub-tropical mountains where conifers belong to different families (Podocarpaceae and Araucariaceae on Kinabalu vs. Pinaceae and Cupressaceae on Yakushima). This suggests the common mechanism of coexistence of shade-intolerant conifers and angiosperm trees in relation to competition for light across a combined gradient of climate and soil. However, there were two types of exceptions to this rule.

First, contrary to our prediction, mixed conifer–angiosperm forests on Kinabalu did not show a more undulating canopy surface (or greater CV of LOCH) than broadleaf forests in the montane zones ≥1560 m a.s.l. (Fig. 6). This implies that some environmental factors limit the height growth of trees, resulting in a relatively smooth canopy surface in spite of the existence of conifers. Kinabalu is situated in the equatorial area (<10 ° latitude) free from tropical cyclones, and trees may suffer less from physical damage by winds compared with those in Yakushima (see later). Instead hydraulic limitation including occasional droughts associated with El Niño events may be responsible for the smooth canopy surface (Aiba *et al.*, 2007). We found that the effects of the 1997–1998 El Niño drought became more severe with increasing altitude on Kinabalu (Aiba and Kitayama, 2002; Aiba *et al.*, 2005, 2010). Moreover, smaller tracheids or vessels caused by slower cambium growth due to year-round low temperature at higher elevations especially on ultrabasic soil may lead to increased hydraulic resistance of the tree stem and thus may limit tree height. In contrast, the existence of hot summers on sub-tropical/temperate mountains may allow the formation of larger tracheids or vessels, even though the mean annual temperature is similar between the upper slope of Kinabalu and Yakushima (Table 1).

Secondly, indistinct upper canopies and undulating canopy surfaces were not necessarily restricted to mixed conifer–broadleaf forests. Two types of broadleaf forests on Kinabalu had similar canopy structures to those of mixed forests. The first type was two lowland broadleaf forests (K07S and K07U) dominated by dipterocarps, which had sparse upper canopies and highly undulating canopy surfaces. The other was a sub-alpine scrub on ultrabasic rock (K31U) dominated by the small-leaved angiosperm *L. recurvum* (Myrtaceae), which had a short, sparse canopy. The possible reasons why these two types of broadleaf forest had a similar canopy structure to that of mixed forests are discussed below.

Tropical lowland broadleaf forests on Kinabalu showed similar canopy structures to montane mixed forests (1050–1200 m) on Yakushima (Fig. 6). In terms of stratification of species (*sensu* Grubb *et al.*, 1963), both forests have a two-storey structure, with dipterocarps (Kinabalu) and conifers (Yakushima) as emergent trees well beyond the general canopy layer (Aiba and Kitayama, 1999; Aiba *et al.*, 2007; Ishii *et al.*, 2010). In terms of stratification of the canopy, there is no clear layer, or these forests are ‘unstratified’ (Koike and Syahbuddin, 1993). The existence of emergent trees is a characteristic of tropical lowland forest of South-east Asia dominated by Dipterocarpaceae, although trees of other families (e.g. Leguminosae and Anacardiaceae) can also become emergents (Whitmore, 1984; Koike and Syahbuddin, 1993; Slik *et al.*, 2010). On Yakushima, lowland broadleaf forests lack the emergent layer, although they had a warmer climate than montane mixed forests (Fig. 4). These facts suggest that, besides environmental factors, the regional species pool is important as a cause of the existence of an emergent layer (Koike and Hotta, 1996).

Given the existence of potentially emergent species in the regional flora, an unstratified canopy would be established if co-occurring species differ in their maximum attainable heights and if the environmental factors do not severely limit the heights of species of taller stature. This hypothesis seems to be satisfied in lowland broadleaf forests in South-east Asia (Koike and Syahbuddin, 1993) and in mixed forests on Yakushima (Ishii *et al.*, 2010). In contrast, many species in broadleaf forests at 30–700 m on Yakushima show similar maximum heights (approx. 10–20 m; Koike and Hotta, 1996; Aiba and Kohyama, 1997), and a monomodal peak of canopy area in broadleaf forests of Yakushima (Fig. 4) may be attributable to the lack of potentially emergent species. On the other hand, strong winds in the summit region of Yakushima, which are related to increased topographic exposure, explain the lower maximum height of emergent conifers as well as the lower maximum stand height in the Y16G plot located almost at the forest limit (Irikura, 1984; Ohsawa, 1984). Mechanical damage caused by strong winds, especially by typhoons, limited the height growth of conifers on Yakushima (Takashima *et al.*, 2009; Ishii *et al.*, 2010). Monolayer structures of dense canopy near the forest limit, where strong winds are the norm, seem to be a general trend worldwide even though such forest or scrub can be a monodominant stand of conifer (Koike *et al.*, 1990).

On Kinabalu, as explained earlier, *L. recurvum* dominates on shallow soils in the summit region at >3300 m on granite (Smith, 1980) as well as in sub-alpine scrub on ultrabasic rock >2700 m such as our K31U plot (Lee and Lowry, 1980; Aiba and Kitayama, 1999). *Leptospermum recurvum* seems to be superior to conifers and other angiosperm trees in the harshest environments across a combined gradient of elevation and soil/substrate conditions on Kinabalu. The lack of small-sized stems in the conifer-dominated forests (K27U and K31G plots; Supplementary Data Fig. S3) indicates that *L. recurvum* is shade intolerant. Furthermore, this species has extremely small leaves (approx. 5 mm long and a surface area of approx. 13 mm^2^; Lee and Lowry, 1980). Based on these facts, *L. recurvum* may be functionally more similar to conifer rather than most angiosperm species. This would explain why the K31U plot (monodominant stand of *L. recurvum*) had forest structure more similar to mixed conifer–broadleaf forests than to other broadleaf forests on Kinabalu (Fig. 6). Angiosperm trees often grow at the tree line around the world, and many of the evergreen species are morphologically similar to conifers such as *L. recurvum* (e.g. *Erica* in East Africa and *Calluna* in Europe) but the deciduous species are not (e.g. *Betula* at the Arctic tree line) (Walter and Breckle, 1986; Körner, 2003). Further study is needed to determine which characters (morphological or physiological) contribute to the adaption of trees to harsh environments at the tree line worldwide.

### Conifer–angiosperm coexistence and additive basal area

Conifer persistence is often associated with large-scale, episodic disturbances (Enright and Ogden, 1995), but we have not found evidence that large-scale disturbance (e.g. fires) is important for tree regeneration in our study sites. Canopy gap formation seems sufficient for regeneration of shade-intolerant conifers (and *L. recurvum*) in most of our study forests, leading to conifer–angiosperm coexistence. At first sight, dense understorey in mixed forests found in the present study (Figs 3 and 4) may seem to prevent the regeneration of conifers when canopy gaps are formed. However, stand-level LAI is small in montane forests on Kinabalu (Kitayama and Aiba, 2002), and the negative effect of relatively high canopy area in the understorey on conifer regeneration would be negligible compared with the positive effect of a sparse upper canopy. Similarly, exceptionally dense canopy at 2–6 m above ground in the K31U plot would not prevent the regeneration of *L. recurvum*, because the vegetation is an open scrub rather than a forest, as is indicated by a very small stand basal area (Fig. 2). On the other hand, tropical lowland forests, despite the sparse upper canopy, have larger LAI than montane forests (Kitayama and Aiba, 2002), and this may hinder the regeneration of shade-intolerant conifers (Coomes and Bellingham, 2011). Temperate evergreen coniferous forests generally have a greater LAI than tropical evergreen broadleaf forests (Asner *et al.*, 2003). This suggests that the clustered distribution of needles does not have as strong shading effects as less clustered broad leaves of tropical forest, allowing the large accumulation of LAI in some conifer stands (Jarvis and Laverenz, 1983).

Among our study sites, the additive basal area of conifers was most pronounced in mixed forests well below the forest limit on Yakushima (Y12Ga and Y12Gb) where conifers became emergent above the broadleaf canopy, but was less clear on Kinabalu (Fig. 2; Aiba *et al.*, 2007). This contradicts the global pattern that, except for Yakushima, additive basal area has been reported exclusively for mixed forests dominated by Araucaricaceae and Podocarpaceae. However, the two forests with the highest stand basal area on Kinabalu (K17UQ and K31G) showed conifer dominance, which may be regarded as an indication of the additive basal area of conifers. The K17UQ plot showed CHP with bimodal peaks, which was similar to that of the mixed forest near the forest limit on Yakushima (Y16G), whereas the K31G plot showed an evenly distributed canopy area (Figs 3 and 4). The upper peak or portion of CHPs of these forests will mostly comprise conifers, judging from the basal area distribution of conifers across diameter classes (Supplementary Data Fig. S3). Thus, CHPs of mixed forests showing an additive basal area (Y12Ga, Y12Gb and, to lesser extents, Y16G, K17UQ and K31G) support the earlier idea that differential utilization of light by taller conifers and shorter angiosperm trees contributes to increased above-ground biomass packing at stand level (Enright, 1982; Ogden, 1985; Enright and Ogden, 1995; Lusk, 2002; Aiba *et al.*, 2007). Narrow crowns of emergent or overstorey conifers will cast little shade on the broadleaf canopy beneath and enable large accumulation of stand basal area (and above-ground biomass). In contrast, the other montane mixed forests on ultrabasic rock of Kinabalu (K17U and K27U) did not show an additive basal area. Above-ground competition may be weak in these forests because of a low LAI (Kitayama and Aiba, 2002), allowing shade-intolerant conifers to codominate with angiosperm trees in the open canopies with little differentiating canopy heights (Aiba *et al.*, 2007). The latter situation may also apply to temperate mixed forest of conifers and deciduous broadleaf trees at high latitudes in the Northern Hemisphere (e.g. *Abies*–*Betula* forest; Koike and Hotta, 1996).

In the above context, it is intriguing that tropical lowland dipterocarp forests of South-east Asia do not show a high basal area in spite of the existence of an emergent layer: the basal area of dipterocarp forest is typically <50 cm^2^ m^−2^ (Slik *et al.*, 2010). As was mentioned earlier, tropical lowland forest, despite its sparse upper canopy and small basal area, has a large LAI composed of broad leaves, and intense competition for light may limit further accumulation of basal area. It should be noted, however, that basal area is not a perfect surrogate of above-ground biomass, which also depends on tree height and wood density. Emergent trees of dipterocarp forests contribute to greater above-ground biomass in Borneo than in the Neotropics (Slik *et al.*, 2010), suggesting that additive effects can be recognized if we examine above-ground biomass. Above-ground competition for light should have been important for the evolution of emergent status for both conifers (competition against angiosperms) and angiosperm trees (competition among angiosperms). Emergent angiosperms in South-east Asian tropics and conifers worldwide usually have wind-dispersed seeds and all conifers are pollinated by wind, which might also have selected for taller statures (Slik *et al.*, 2010).

### Conclusions

The portable LIDAR system of Parker *et al.* (2004) used in this study is a powerful tool to characterize forest structure along altitudinal gradients on both tropical and sub-tropical mountains. It successfully reproduced a general pattern of maximum tree height across altitude even for such lofty forests >50 m high (Fig. 1). Earlier studies using the same system examined forest structure in a successional gradient or in different logging-disturbance regimes under the same climate (Parker *et al.*, 2004; Akutsu *et al.*, 2007). Our study demonstrated the utility of the system for the comparison across an altitudinal gradient equivalent to the climatic change from equatorial to cool-temperate forests. For example, it revealed the unexpected finding that sub-tropical mixed conifer–broadleaf forest and tropical lowland broadleaf forest had similar canopy structures (Fig. 6). However, this system (and LIDAR in general) cannot separate the leaf and other organs (e.g. stems and branches) or leaves of different species (e.g. conifers and angiosperms). More time-consuming and labour-intensive methods will be required to study the vertical distribution of leaves and to distinguish leaves of different species (e.g. Kato *et al.*, 1978; Fukushima *et al.*, 1998; Sumida *et al.*, 2009).

In summary, this study suggests that shade-intolerant conifers can coexist with angiosperm trees in cold and nutrient-poor environments, either as emergents above the closed canopy of angiosperm trees or as codominants in the open canopy. In contrast, competitive exclusion by angiosperm trees may explain the absence of conifers in warm and nutrient-rich environments. Therefore, the outcome of competition for light between conifers and angiosperms varies along environmental gradients when other environmental factors such as climate and soils strongly limit angiosperm growth.

## SUPPLEMENTARY DATA

Supplementary data are available online at www.aob.oxfordjournals.org and consist of the following. Figure S1: maps showing the study sites. Figure S2: relative canopy height profiles for 2 and 10 m bins across altitudinal gradients on Mount Kinabalu and Yakushima Island. Figure S3: basal area distribution across diameter classes for conifers, *Leptospermum recurvum* (Myrtaceae, on Kinabalu only) and other angiosperm trees in the study plots on Mount Kinabalu and Yakushima Island.

Supplementary Data
