# Supplementary material for: Canopy structure of tropical and sub-tropical rain forests in relation to conifer dominance analysed with a portable LIDAR system
Source: Ann Bot. 2013 Nov 5;112(9):1899–909. doi: 10.1093/aob/mct242 (PMC3838564; doi:10.1093/aob/mct242)
Supplement: Supplementary Data [file supp_112_9_1899__index.html]

Canopy structure of tropical and sub-tropical rain forests in relation to conifer dominance analysed with a portable LIDAR system — Canopy structure of tropical and sub-tropical rain forests in relation to conifer dominance analysed with a portable LIDAR system — Supplementary Data 

# Canopy structure of tropical and sub-tropical rain forests in relation to conifer dominance analysed with a portable LIDAR system

## Supplementary Data

Supplementary Data

**Files in this Data Supplement:**

- Supplementary Data - Pdf file
