# Supplementary material for: Canopy structure of tropical and sub-tropical rain forests in relation to conifer dominance analysed with a portable LIDAR system
Source: Ann Bot. 2013 Nov 5;112(9):1899–909. doi: 10.1093/aob/mct242 (PMC3838564; doi:10.1093/aob/mct242)

SUPPLEMENTARY DATA

FIG. S1. Maps showing the study sites: (A) a map of east Asia showing the locations of Yakushima Island, Japan and Mount Kinabalu, Malaysia (https://maps.google.com/), (B) a map of Yakushima, and (C) a map of Kinabalu. Contour lines, distribution of geological substrates and the locations of the study plots are shown in (B) and (C). See Table 1 for the site names.

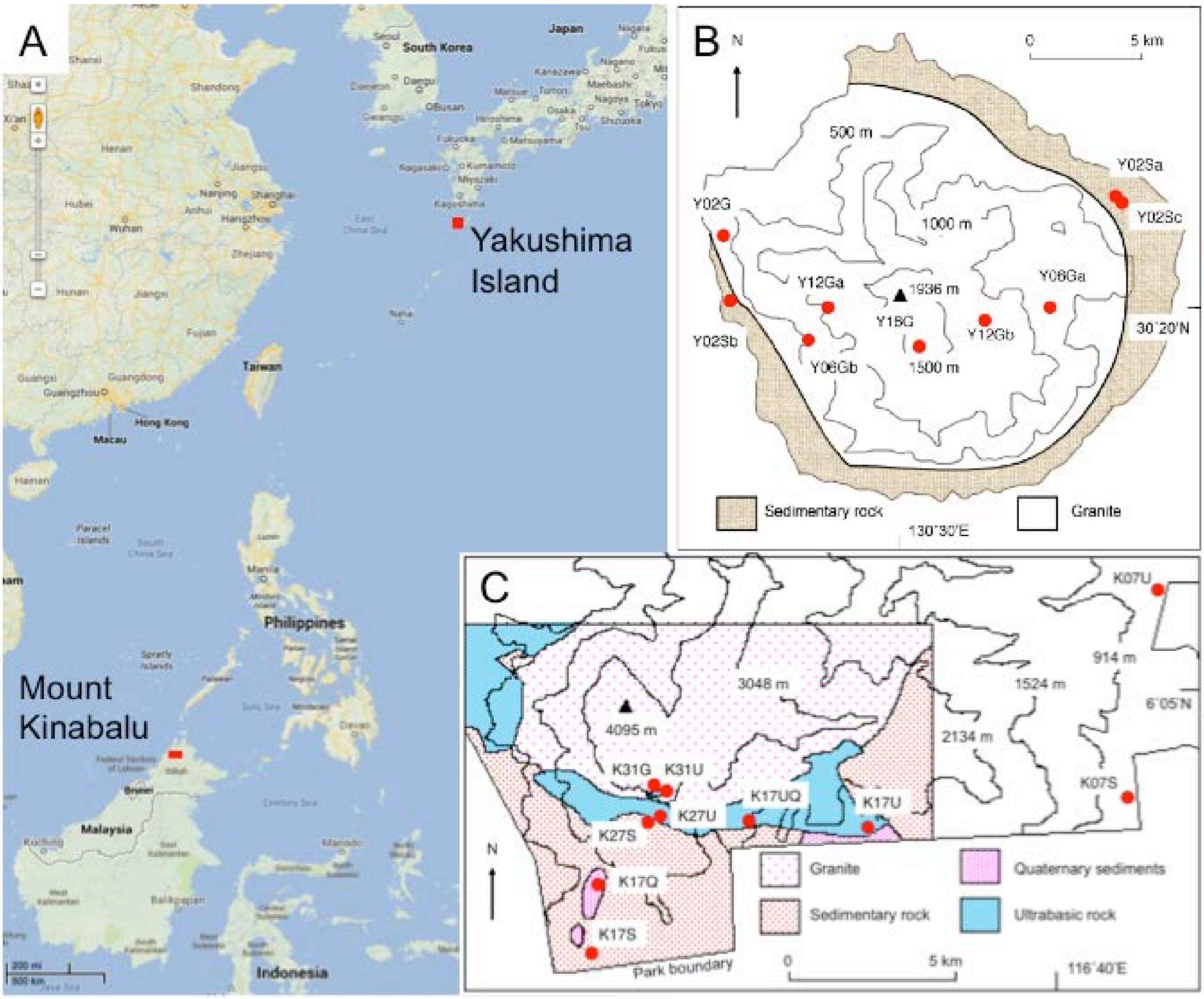

FIG. S2. Relative canopy height profiles across altitudinal gradients on: (A) Mount Kinabalu for 2-m bins, (B) Mount Kinabalu for 10-m bins, (C) Yakushima Island for 2-m bins, and (D) Yakushima Island for 10-m bins. See Fig. 3 for the explanation of figure.

A

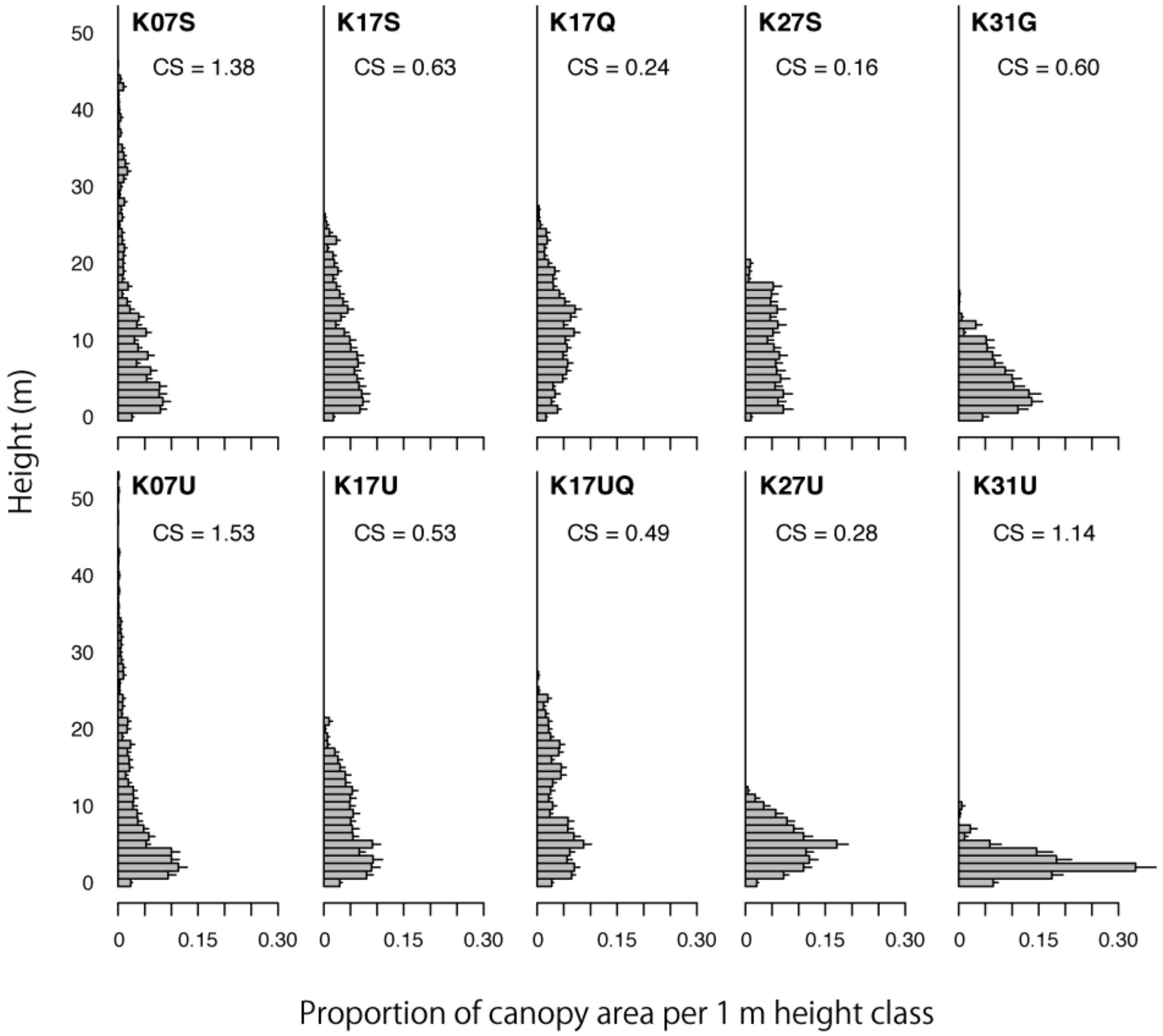

FIG. S2. Continued.

B

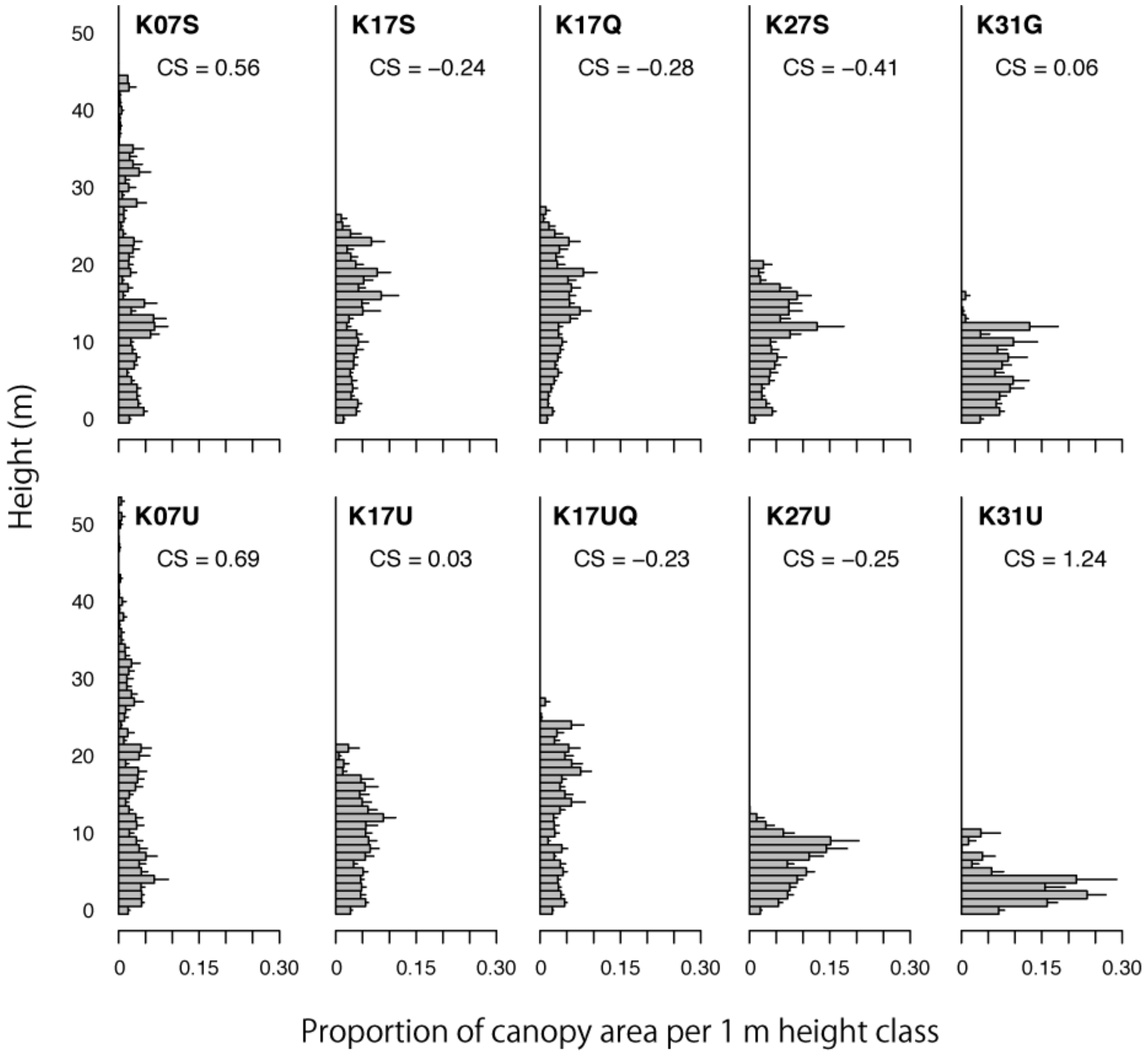

FIG. S2. Continued.

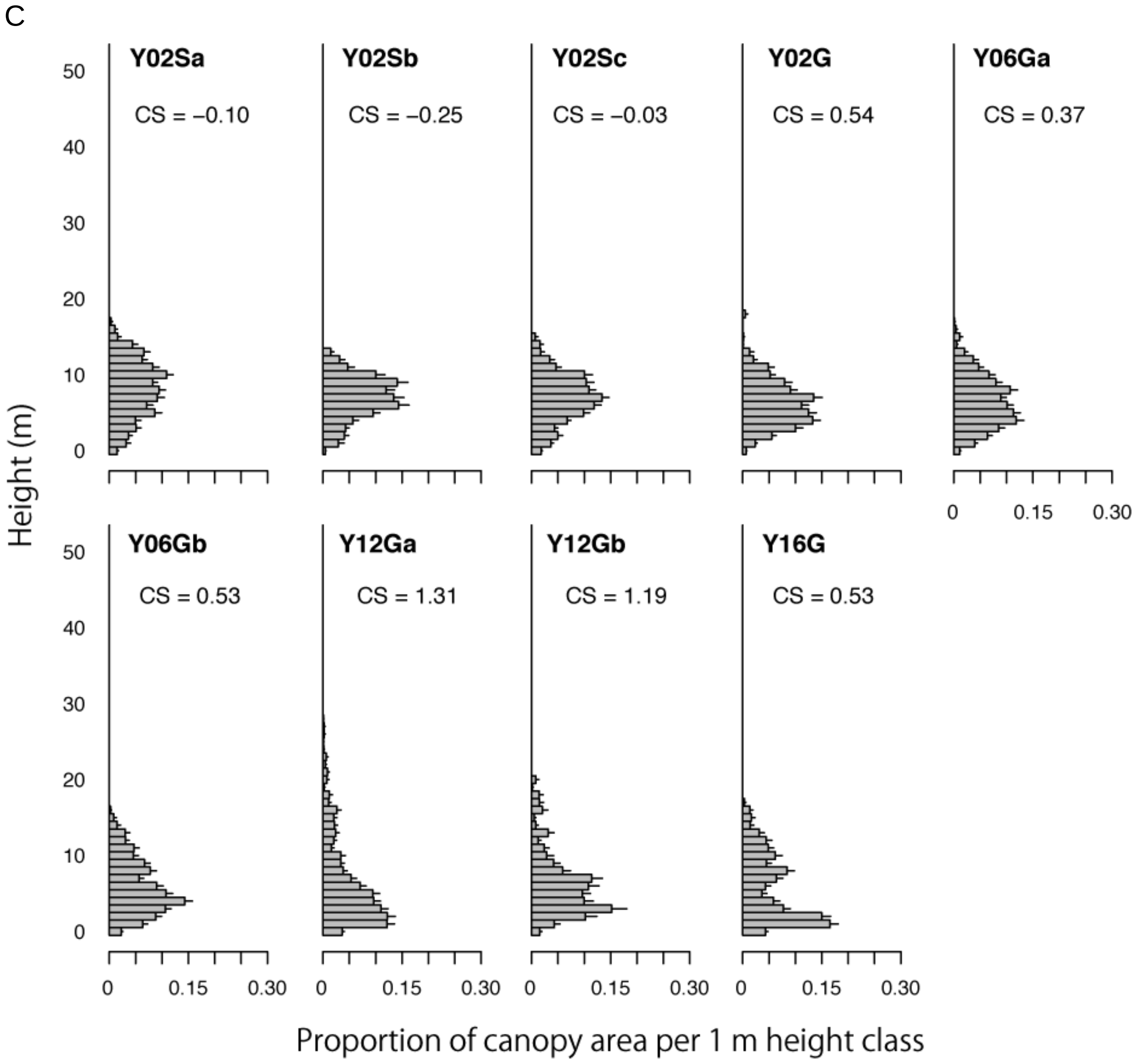

FIG. S2. Continued.

D

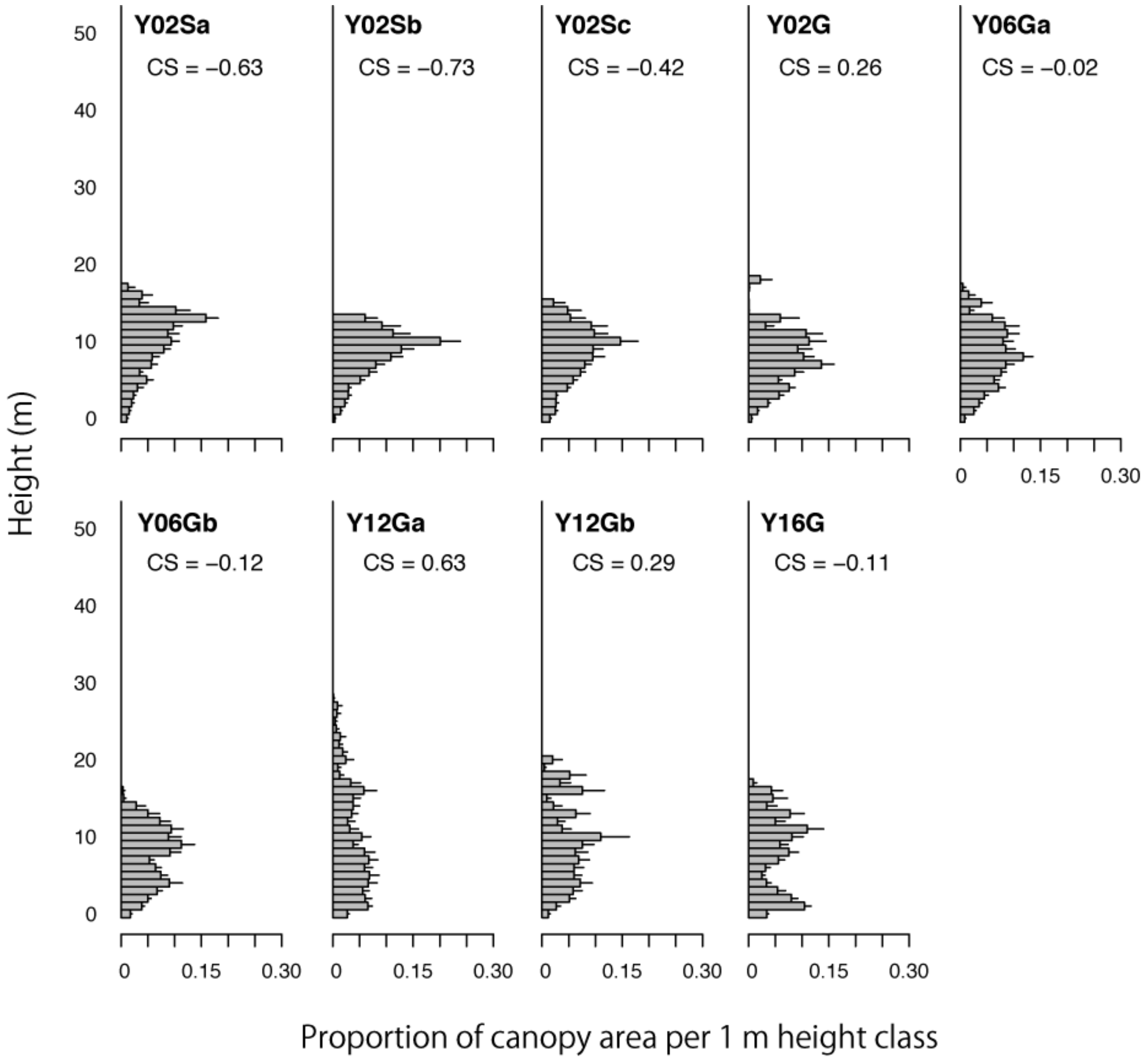

FIG. S3. Basal area distribution across diameter classes for conifers, *Leptospermum recurvum* (Myrtaceae, on Kinabalu only) and other angiosperm trees in the study plots on: (A) Mount Kinabalu and (B) Yakushima Island. See Table 1 for site names.

A

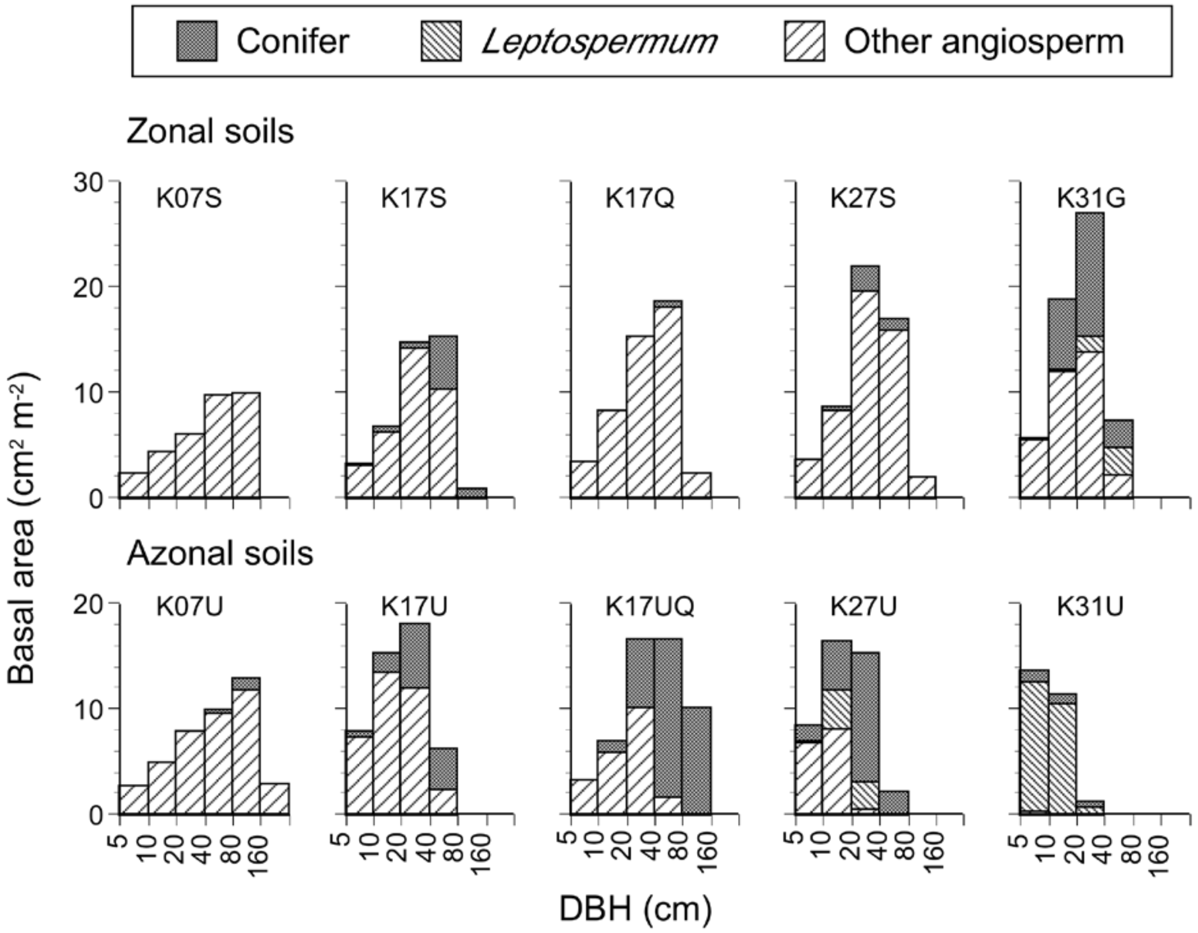

FIG. S3. Continued.

B

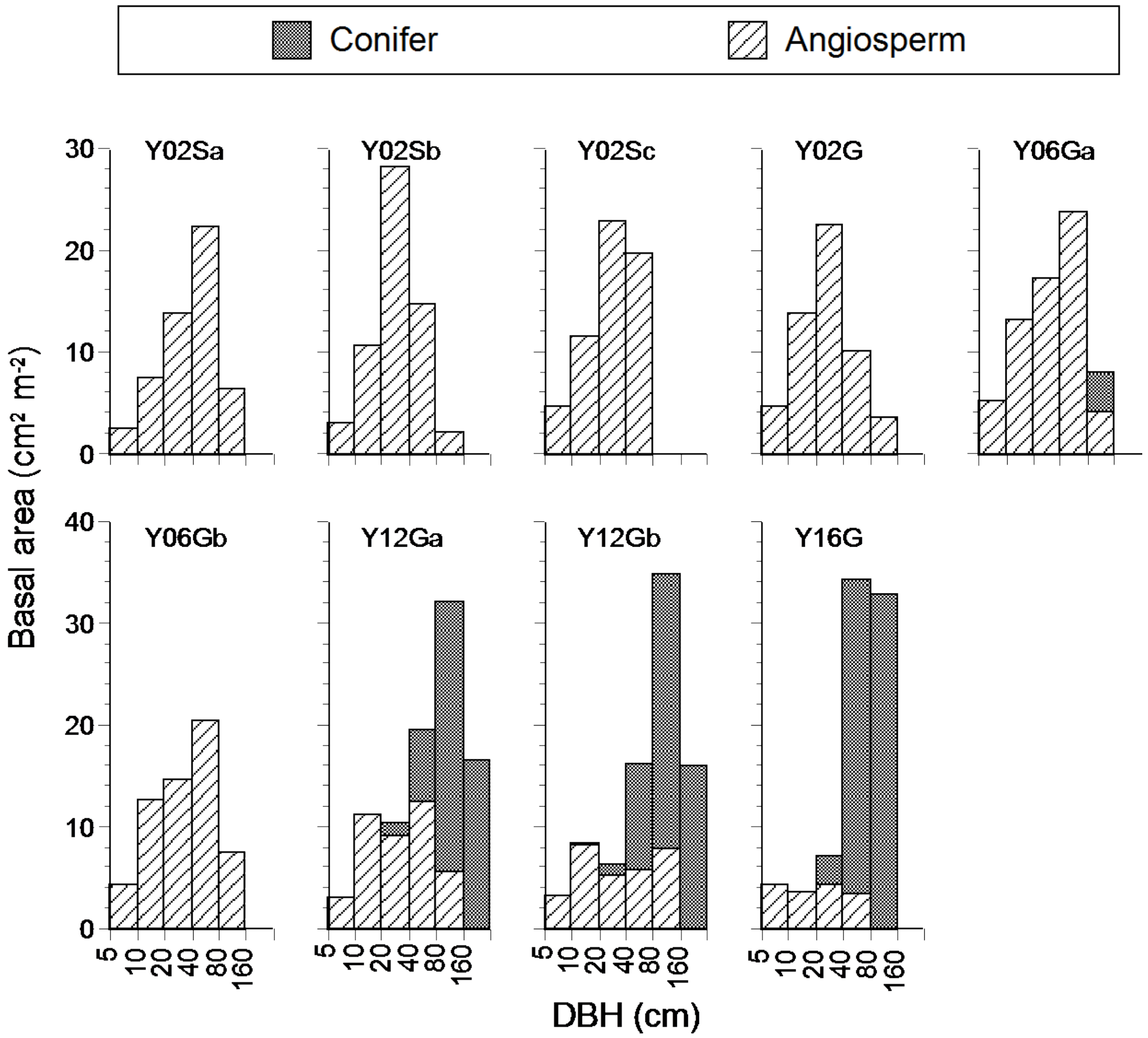

Supplement: Supplementary Data [file supp_mct242_mct242supp.pdf]
